# Do wind energy installations alter gastrointestinal parasite infection patterns in roe deer (*Capreolus capreolus* L.) in central Poland?

**DOI:** 10.1016/j.ijppaw.2026.101300

**Published:** 2026-10-08

**Authors:** Anna Maria Pyziel, Kateryna Slivinska, Rusłan Sałamatin, Joanna Werszko, Marta Kloch, Marcin Świątek, Olena Zhytova, Daniel Klich

**Affiliations:** a Department of Public Health Protection and Animal Welfare, Faculty of Biological and Veterinary Sciences, Institute of Veterinary Medicine, Nicolaus Copernicus University in Toruń, Toruń, 87-100, Poland; b Museum and Institute of Zoology of the Polish Academy of Sciences, Warsaw, 00-818, Poland; c I.I. Schmalhausen Institute of Zoology of the National Academy of Sciences of Ukraine, Kyiv, 01030, Ukraine; d Department of General Biology and Parasitology, Medical University of Warsaw, Warsaw, 02-004, Poland; e Department of Microbiology and Parasitology, Faculty of Medical Science, Collegium Medicum, Cardinal Stefan Wyszyński University in Warsaw, Warsaw, 01-938, Poland; f Department of Animal Genetics and Conservation, Faculty of Bioeconomy and Food Systems, Warsaw University of Life Sciences, Warsaw, 02-787, Poland; g Department of Animal Breeding and Nutrition, Faculty of Bioeconomy and Food Systems, Warsaw University of Life Sciences, Warsaw, 02-787, Poland; h Polissia National University, Zhytomyr, 10008, Ukraine

**Keywords:** Renewable energy, Wildlife health, Ruminants, Nematodes, Cestodes

## Abstract

The European roe deer (*Capreolus capreolus* L.) is a widespread cervid in Europe and an important reservoir of gastrointestinal helminths. Although environmental and host-related drivers of parasite communities have been extensively studied, the effects of anthropogenic infrastructure on host-parasite interactions remain poorly understood. This study aimed to characterize gastrointestinal helminth diversity in roe deer from central Poland and to evaluate the influence of wind farms on parasite infections. A total of 102 roe deer collected between 2022 and 2024, hunted in accordance with local management plans, were examined for helminths in the abomasum, small intestine, and large intestine. The effects of wind farm presence on parasite abundance were assessed using generalized linear models with a negative binomial distribution. The gastrointestinal helminth community was diverse, comprising seven species in the abomasum, seven in the small intestine, and five in the large intestine. *Haemonchus contortus* dominated the abomasal community, whereas *Chabertia ovina* was the most prevalent species in both intestinal sections. Tapeworms of the genus *Moniezia* were additionally detected in the small intestine of four animals. With the exception of *Ch. ovina* and *Trichuris* sp., helminths were generally more abundant in roe deer from wind farm areas, which also harbored greater species richness than animals from control areas. Wind farm presence was a significant predictor in four of seven models explaining nematode abundance, with nematodes consistently more abundant in wind farm areas. Sex was included in most models, indicating lower nematode abundance in females than in males, whereas study area and age were retained only in selected models and showed less consistent effects. Overall, these findings indicate differences in gastrointestinal helminth communities between roe deer inhabiting wind farm and control areas and suggest that the presence of wind farms may be associated with variation in parasite infections.

## Highlights

• A total of 102 roe deer on wind farms and control areas were examined.
• Helminths were recovered from the abomasum, small intestine, and large intestine.
• Wind farm significantly predicted abomasum and small intestine nematode numbers.
• *Haemonchus contortus* and *Trichuris* sp. presented higher numbers on wind farms.

## Graphical abstract

**Figure:**
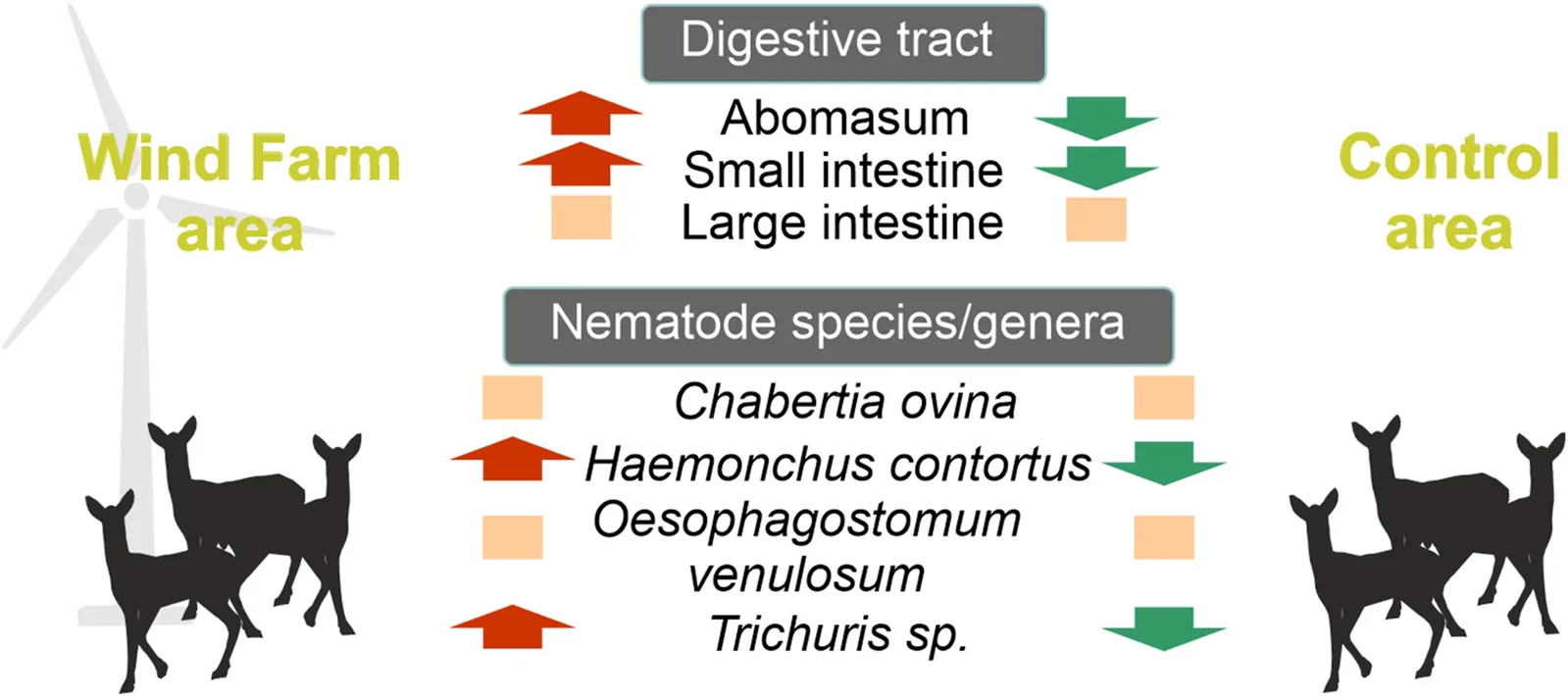

## 1 Introduction

The European roe deer (*Capreolus capreolus*) is one of the most abundant wild ungulates in Europe, including Poland, where its population has been steadily increasing in recent decades (Apollonio et al., 2010; Putman et al., 2011). As a widely distributed species inhabiting agricultural and forest mosaics, roe deer play an important ecological role. At the same time, they act as reservoirs and transmitters of numerous parasites, including gastrointestinal helminths of both veterinary and ecological significance (Gortázar et al., 2007; Morgan et al., 2013).

Gastrointestinal nematodes (GINs) of ruminants belong to six families: Trichostrongylidae Leiper, 1912 (genera: *Ostertagia, Spiculopteragia, Mazamastrongylus, Trichostrongylus, Haemonchus, Ashworthius*), Molineidae Skrjabin and Schultz, 1937 (genus *Nematodirus*), Capillariidae Neveu-Lemaire, 1936 (genus *Aonchotheca*), Trichuridae Baird, 1853 (genus *Trichuris*), Ancylostomatidae Lane, 1907 (genus *Bunostomum*), and Chabertidae Popova, 1952 (genera *Chabertia* and *Oesophagostomum*) (Hoberg et al., 2001). Representatives of the Trichostrongylidae and Molineidae belong to the superfamily Trichostrongyloidea, whereas those of Capillariidae and Trichuridae are classified to Trichinelloidea, and those of Chabertidae to Strongyloidea (Marchiondo et al., 2019a, 2019b; Snyder et al., 2019). These nematodes occupy different regions of the host's digestive tract: many representatives of the Trichostrongylidae occur predominantly in the abomasum, whereas members of the Molineidae, Capillariidae, and Ancylostomatidae are typically associated with the small intestine. In contrast, nematodes of the families Trichuridae and Chabertidae predominantly inhabit the large intestine (Bolukbas et al., 2012; González et al., 2023).

Recent large-scale syntheses indicate that cervids in Europe harbor a wide range of generalist nematodes with broad host spectra, including species commonly associated with livestock, such as *Haemonchus contortus, Ostertagia ostertagi, Trichostrongylus vitrinus, T. axei, T. capricola, Oesophagostomum venulosum,* and *Chabertia ovina*, among others (Winter et al., 2018). Notably, it has been demonstrated that some abomasal nematodes of cervids can be transmitted to domestic ruminants, including anthelmintic-resistant strains of *H. contortus* (Chintoan-Uta et al., 2024). This underscores the epidemiological importance of parasite exchange between wild ruminants and livestock (Brown and Morgan, 2024). Consequently, such transmission can adversely affect the profitability of animal production, particularly in infections with blood-feeding helminths, such as *H. contortus* and *A. sidemi*, which may cause haemorrhagic anaemia in the host (Gilleard, 2013; Kołodziej-Sobocińska et al., 2016).

A combination of environmental, ecological, and host-related factors shapes helminth communities in roe deer. Among these, habitat type has been identified as a key determinant of abomasal nematode community composition, with distinct parasite assemblages associated with forest and agricultural environments (Wyrobisz-Papiewska et al., 2025). More broadly, anthropogenic changes are increasingly recognized as major drivers of host-parasite dynamics (Bradley and Altizer, 2007). While their ecological impacts on wildlife behavior and habitat use have been widely documented (Köppel, 2017), their effects on wildlife health remain less well understood.

In this context, recent studies suggest that wind turbines may act as chronic stressors for terrestrial mammals, potentially affecting their physiology and immune responses (Klich et al., 2020; Teff-Seker et al., 2022). The potential impact of wind farms on terrestrial mammal species, however, can appear in a much broader context. In previous studies, a reduction in the use of movement corridors and grazing habitats and increased fragmentation of calving ranges have been observed (e.g., Skarin et al., 2015, 2018). Often, the effect was observed as avoidance of wind farm interiors and direct proximity to turbines (e.g., Łopucki et al., 2017; Kim et al., 2025; Kumara et al., 2022) or as increased vigilance and anti-predator behavior (Rabin et al., 2006). Consequently, the overall abundance of certain species in areas with wind farms is likely to be affected (Klich et al., 2024). It should be noted that many other studies did not report negative effects of this infrastructure on terrestrial mammals (e.g., Flydal et al., 2004; Walter et al., 2006; Taylor et al., 2016), suggesting that responses can be species- and context-dependent (Schöll and Nopp-Mayr, 2021). Therefore, the effect, if not directly shown, may be later observed as an indirect effect (e.g., Sirén et al., 2017; Gmaj and Klich, 2026).

Despite growing interest in wildlife parasitology, the potential influence of wind farm infrastructure on gastrointestinal parasite communities in wild ungulates remains unexplored. This gap in knowledge highlights the need for studies addressing how renewable energy development, such as wind farms, may influence wildlife health.

Therefore, this study aimed to: characterize the diversity and prevalence of gastrointestinal helminths in roe deer from central Poland; assess spatial variation in parasite communities across different regions and sections of the digestive tract; and evaluate the potential impact of wind farm presence on helminth species composition and prevalence.

## 2 Material and methods

### 2.1 Study area

European roe deer samples were collected in Central Poland, within the Mazowieckie and Łódzkie voivodeships, across three sites: Iłża (51°10′7″N, 21°11′0″E), Rawa Mazowiecka (51°48′22″N, 20°4′30″E), and Węgrów (52°23′3″N, 21°50′0″E) (Fig. 1). These areas, part of the Central European Plain, are characterized by predominantly flat terrain with an average elevation of 173 m above sea level (Łowicki and Mizgajski, 2013). Shaped by glacial processes, the landscape comprises diverse landforms and soils-from fertile loams to sandy substrates-affecting vegetation structure and microhabitat availability for intermediate parasite hosts. Major rivers, including the Vistula in Mazowieckie voivodeship, create moist riparian zones that favor the survival and development of helminth eggs and larvae.

**Fig. 1:**
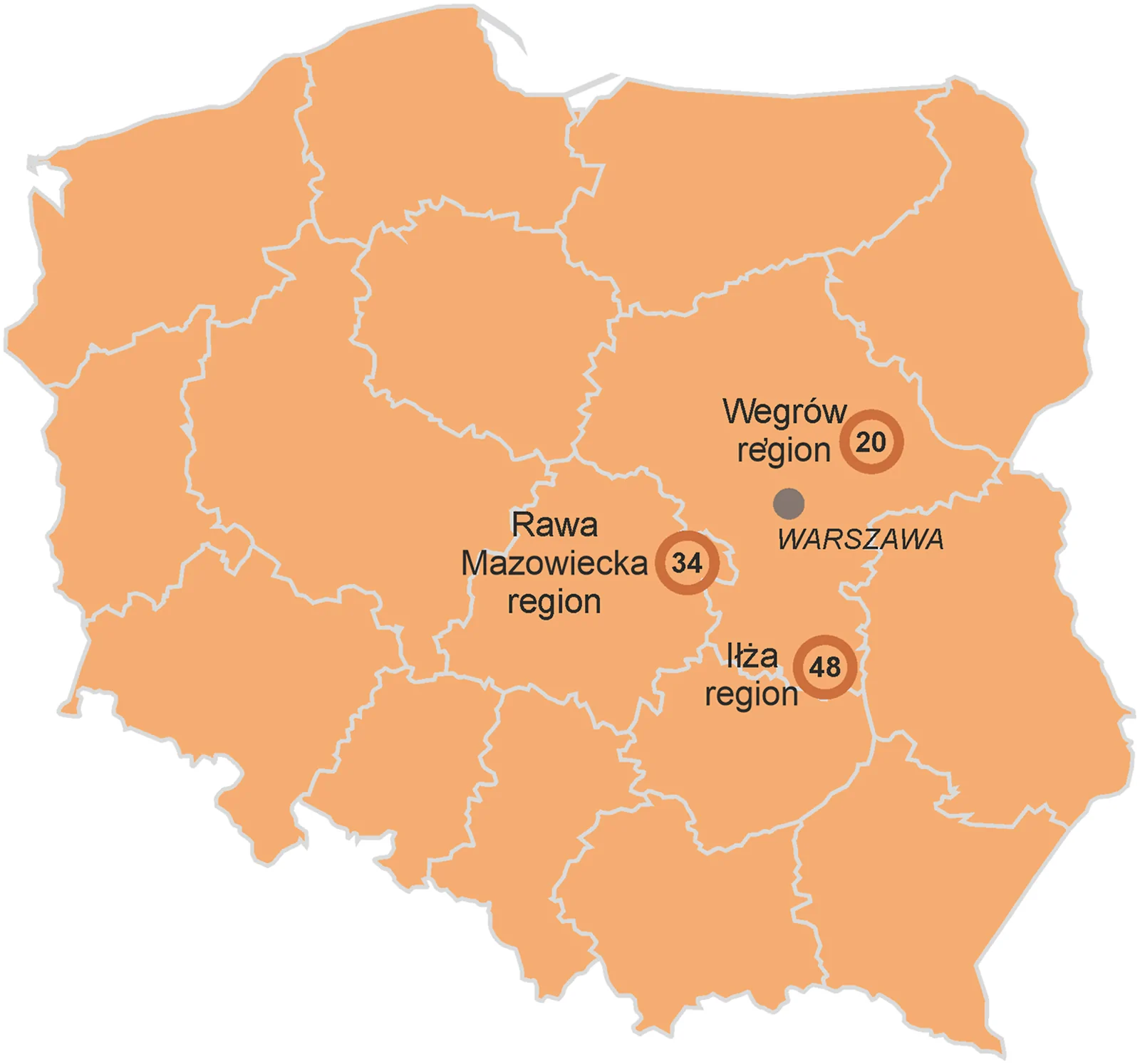
Location of study areas in Poland with the number of obtained roe deer samples.

The temperate climate, with cold winters, warm summers, and peak precipitation during the growing season (Ustrunul et al., 2021), further influences the seasonal dynamics of gastrointestinal parasites, resulting in a heterogeneous environment conducive to parasite persistence and transmission in wild ruminants.

All three sites contain wind farms within agricultural landscapes that partially overlap the hunting grounds where roe deer were sampled. The wind farm at the Iłża site, with a total capacity of about 69 MW, comprises 32 turbines (80–95 m hub height), constructed in 2013–2014 (27 turbines), with five turbines added in 2024. In Węgrów site, there were 39 turbines (126 - 132 m hub height) with a total capacity of about 133 MW (25 built in 2015 and 14 added in 2022-2023). In the Rawa Mazowiecka study area, a total of 30 wind turbines (of 95-105m hub height) of total capacity of about 60 MW were present, of which 20 were constructed between 2012 and 2017, and an additional 10 were installed in 2024. In all areas apart from roe deer, other ungulate species were present: red deer, wild boar, and moose. Among large predators, only wolves were observed; however, their presence was rather sporadic, and no detailed data on wolf activity were recorded. To our knowledge, there are no large numbers of grazing cattle in the surveyed areas. In particular, the wind farm areas did not contain pastures that were used for grazing cattle. While small numbers of cattle, unobserved by us, might have been present in the control areas, the study site itself consisted of a typical mosaic of arable land and forests. Based on roe deer position relative to the turbines, animals were categorized as occurring inside or outside the wind farm. The wind farm area was defined as the space between turbines forming the cluster, plus a 700 m buffer zone. Such a distance is considered safe for humans, at which the wind farm does not have a significant impact (Mroczek et al., 2012). A 700m distance was previously used to assess wind turbine impacts on terrestrial mammals, including roe deer (Klich et al., 2024). Such a distance has been confirmed to influence roe deer occurrence around wind turbines (Łopucki et al., 2018). The roe deer is an animal with a small home range size (e.g. (Kjellander et al., 2004; Lovari et al., 2017), suggesting that individuals encountered within the designated buffer were likely under the constant, or at least partial, influence of wind farms, although it is possible that some of them were present in the zone of influence only temporarily.

### 2.2 Examined animals

Hunting management was actively practiced in the study areas, with licensed hunters harvesting a predetermined number of game animals, including roe deer, each year in compliance with Polish hunting regulations and management plans. Roe deer hunting is divided into two seasons. Bucks are hunted from 11 May to 30 September, while does and fawns are hunted from 1 October to 15 January (though samples were collected during November and December). Consequently, samples from females represent the winter period, whereas samples from males represent the summer period. Between 2022 and 2024, samples were collected in collaboration with local hunting clubs under formal agreements. All samples were obtained as by-products of routine hunting activities. For each individual, metadata including sex, age, date, and location of acquisition were recorded. Hunters provided the entire digestive tract post-mortem, which was then transported to the laboratory for parasitological analysis.

The sampling strategy aimed to ensure that animals originated from comparable environmental conditions, agricultural landscapes of Central Poland, and experienced similar hunting pressure. In total, 102 roe deer were sampled: 39 from wind farm areas and 63 from outside these areas (Fig. 1).

Individual sections of the digestive tract, including the abomasum, small intestine, and large intestine, were ligated and separated from each animal. In some instances, specific sections could not be collected due to damage (Table 1). Afterward, they were brought to the laboratory and frozen.

**Table 1:** The number of samples from roe deer inhabiting areas outside the wind farms (C – control), and inside the wind farms (F – farm) in various regions of Central Poland.

Sample	Iłża region	Rawa Mazowiecka region	Węgrów region	Together
Animals examined	48 (30 C, 18 F)	34 (22 C, 12 F)	20 (11 C, 9 F)	102 (63 C, 39 F)
Abomasum	48 (30 C, 18F)	31 (21 C, 10 F)	19 (11 C, 8 F)	98 (62 C, 36 F)
Small intestine	46 (29 C, 17 F)	33 (21 C, 12 F)	19 (10 C, 9 F)	98 (60 C, 38 F)
Large intestine	43 (27 C, 16 F)	23 (15 C, 8 F)	19 (10 C, 9 F)	85 (52 C, 33 F)

### 2.3 Collection of helminths

Collected sections of the gastrointestinal tract were thawed before processing. In each case, the contents of the abomasum, small intestine, and large intestine (colon and cecum) were separately mixed with tap water at a 1:10 ratio, decanted several times, and washed through a sieve (mesh size: 100–150 μm). The resulting sediment was examined for parasites using a Delta Optical SZ-450 T stereomicroscope (Delta Optical, Mińsk Mazowiecki, Poland). Collected parasites were placed in tubes containing 70% ethanol for subsequent morphological and molecular analyses.

### 2.4 Morphological examination

The anterior and posterior parts of the nematode specimens were excised and transferred into a drop of lactophenol on a microscope slide to render the cuticle transparent and to allow visualization of the oral cavity of males and females and spicules of males (Pyziel-Serafin et al., 2023). The middle body parts of the examined specimens were individually transferred into Eppendorf tubes containing 70% ethanol and appropriately labelled.

Species identification of the representatives of the superfamily Trichostrongyloidea was based on the morphology of the male posterior ends. Thus, their female specimens were not included in the study. The identification of “small” nematodes of the subfamily Ostertagiinae was based on their spicules (Dróżdż, 1995); whereas the identification of “large” abomasal nematodes of the subfamily Haemonchinae was based on the symmetry of the copulatory bursa and position of the dorsal lobe (Lehrter et al., 2016). Namely, *H. contortus* is characterized by an asymmetrical copulatory bursa with a dorsal lobe directed to the left, which possesses a Y-shaped dorsal ray, angled relative to the longitudinal axis of the spicules; whereas *A. sidemi* has a symmetrical copulatory bursa with a dorsal lobe in a central position (Naaz et al., 2025). In turn, specimens of the Trichinelloidea were identified to the genus level as *Aonchotheca,* characterized by a slender, thread-like body, or *Trichuris* (whipworm), characterized by a long, thin anterior end and shorter, thicker posterior end, with a whip-like appearance (Marchiondo et al., 2019b). Small intestine nematodes of the genus *Bunostomum* were identified based mainly on the morphology of the anterior end of both male and female specimens, with *B. phlebotomum* characterized by a pair of chitinous cutting plates on the ventral margin of the buccal capsule and the absence of dorsal teeth, and *B. trigonocephalum* characterized by the presence of a dorsal tooth extending from the base of the buccal cavity anteriorly towards the mouth opening (Marchiondo et al., 2019a; Wilfred and Lee, 1981). Similarly, representatives of the family Chabertidae were identified based on the morphology of the buccal capsule. *Chabertia ovina* (large-mouth bowel worm) has a large bell-shaped buccal capsule with an oval aperture surrounded by a double row of small papillae around the rim (Marchiondo et al., 2019a), whereas *Oe*. *venulosum* was identified by its shallow, cylindrical, and toothless buccal capsule and by the mouth opening surrounded by a cuticular collar bearing a leaf crown composed of an external and an internal corona radiata (Gaddam et al., 2017; Marchiondo et al., 2019a). Additionally, the length of 35 spicules of *Chabertia* sp. males was measured.

Species identification of the nematodes was performed using a LAB40 light microscope (OPTA-TECH, Warsaw, Poland) at magnifications of 100×-400×. Microphotographs were captured using a digital camera, and measurements were obtained with OPTA View-15 software (OPTA-TECH). Due to the poor preservation of the cestode specimens, morphometric analyses could not be conducted; therefore, molecular methods were applied instead.

### 2.5 DNA extraction, amplification, and sequencing

#### 2.5.1 Nematodes

Genomic DNA was individually extracted from 50 intestinal nematodes to enable molecular species identification (25 individuals of *Oesophagostomum* sp. and 25 of *Chabertia* sp.) using a NucleoSpin Tissue DNA extraction kit (Macherey-Nagel, Düren, Germany), following the manufacturer's protocol.

Partial regions of the internal transcribed spacer 2 (ITS2) and the large subunit (LSU) ribosomal RNA gene were amplified by polymerase chain reaction (PCR) using two oligonucleotide primers: forward NC1 (5′-ACG TCT GGT TCA GGG TTG TT-3′) and reverse NC2 (5′-TTA GTT TCT TTT CCT CCG CT-3′) (Gasser et al., 1993).

PCR amplification was performed in a total reaction volume of 50 μL, containing 20 μL of molecular biology grade water (Sigma-Aldrich, St. Louis, MO, USA), 25 μL of AccuStart II PCR ToughMix (2× concentration) (Quantabio, Beverly, MA, USA), 1 μL of GelTrack Loading Dye (50× concentration) (Quantabio), 1 μL of each primer (20 mM), and 2 μL of template DNA. Negative (molecular biology grade water) control was included in each run.

Thermal cycling conditions followed those described previously (Werszko et al., 2024). The resulting sequences were assembled into contigs using CodonCode Aligner version 8.0 (CodonCode Corporation, Centerville, MA, USA). The obtained nucleotide sequences were compared with those available in the NCBI database using the Basic Local Alignment Search Tool (BLAST) and subsequently submitted to GenBank.

#### 2.5.2 Cestodes

The tissue samples of two tapeworms recovered from the small intestine of roe deer originating from the Węgrów and Iłża regions were rinsed with phosphate-buffered saline (PBS) to remove residual ethanol. Genomic DNA was extracted using the Genomic Mini Kit (A&A Biotechnology, Gdańsk, Poland) in accordance with the manufacturer's instructions.

PCR amplification was performed using primers JB3 (5′-TTTTTTGGGCATCCTGAGGTTTAT-3′) and JB4.5 (5′-TAAAGAAAGAACATAATGAAA-ATG-3′) (Bowles et al., 1993) to obtain a 396 bp fragment of the mitochondrial cytochrome *c* oxidase subunit 1 (cox1) gene.

Each PCR reaction was carried out in a total volume of 25 μL, containing 17.5 μL of deionized water, 0.25 μL of Allegro Taq DNA polymerase (5 U/μL) (Novazym, Poznań, Poland), 0.25 μL of dNTP mix (10 mM), 2.5 μL of 10× Taq DNA polymerase buffer (containing 25 mM MgCl_2_), 0.25 μL of each primer (20 pmol/μL), and 4 μL of template DNA. In the negative control, nuclease-free water was used instead of template DNA.

PCR conditions followed the protocol described previously (Bowles et al., 1993). Amplification products were visualized on 1% agarose gels stained with SimplySafe™ (EURx, Gdańsk, Poland). The gels were documented using a ChemiDoc MP system and Image Lab software (Bio-Rad, Hercules, CA, USA).

The PCR products were purified using the Agarose-Out DNA Purification Kit (EURx, Gdańsk, Poland) and subsequently sequenced by Genomed (Warsaw, Poland). The resulting nucleotide sequences were assembled using CLC Main Workbench version 6.9.1 and analyzed with NCBI BLAST (Sayers et al., 2019). Finally, the sequences were submitted to GenBank.

### 2.6 Phylogenetic reconstruction of cestodes

The 62 nucleotide sequences showing the highest similarity in the NCBI BLAST analysis were selected for phylogenetic tree reconstruction using Bayesian inference implemented in MrBayes version 3.2.7 on the CIPRES Science Gateway (Miller et al., 2010). The sequence of *Echinococcus canadensis* (GenBank: LC184604) was used as an outgroup.

### 2.7 Statistical analysis of the impact of selected factors on the number of nematodes

We used a generalized linear model with a negative binomial distribution and a log link function to evaluate the potential impact of selected factors on the total number of nematodes in different sections of the roe deer digestive tract and on the number of nematodes of selected species/genera in the whole digestive tract. Negative binomial models are suitable for handling overdispersion in count data (Zuur et al., 2009). Three separate models (Models 1-3) were constructed for each section (abomasum, small intestine, large intestine) to account for differences in the total abundance of nematodes. We also constructed four separate models (Models 4-7) for the most abundant nematode species/genus: *Ch. ovina, H*. *contortus, Oe*. *venulosum*, *and Trichuris*.

In each model, the dependent variable was the total number of nematodes in a given section of the digestive tract (for Models 1-3) or the number of nematodes of a given species in the whole digestive tract. Four explanatory variables were included: sex and age of the animals, study area, and the presence of roe deer within wind farm areas. The inclusion of the latter variable was motivated by the potential impact of wind farm infrastructure on roe deer stress levels and health (Klich et al., 2024; Kliczkowska et al., 2025). The effects of the selected factors on nematode abundance were assessed using a model selection approach. Specifically, all possible model variants (i.e., all combinations of explanatory variables), including the null model, were fitted to identify the best-supported model (Burnham and Anderson, 2002). Models were ranked according to the Akaike Information Criterion (AIC), with the model showing the lowest AIC value considered the best-fitting. Only the results of the highest-ranked models were presented.

All statistical analyses were performed using IBM SPSS Statistics 29.0 (Armonk, New York).

## 3 Results

### 3.1 Abomasal nematodes

A total of seven nematode species were identified in the abomasum of roe deer in central Poland (Table S1; Fig. 2). The dominant species was the blood-sucking *H*. *contortus*, which was commonly found in roe deer across all examined regions. The prevalence of *H. contortus* ranged from 35% in the Iłża region to 53% in the Węgrów region. Moreover, this species exhibited the highest infection intensity across all regions, with a maximum of 2558 individuals recorded on a single host in the Węgrów region.

**Fig. 2:**
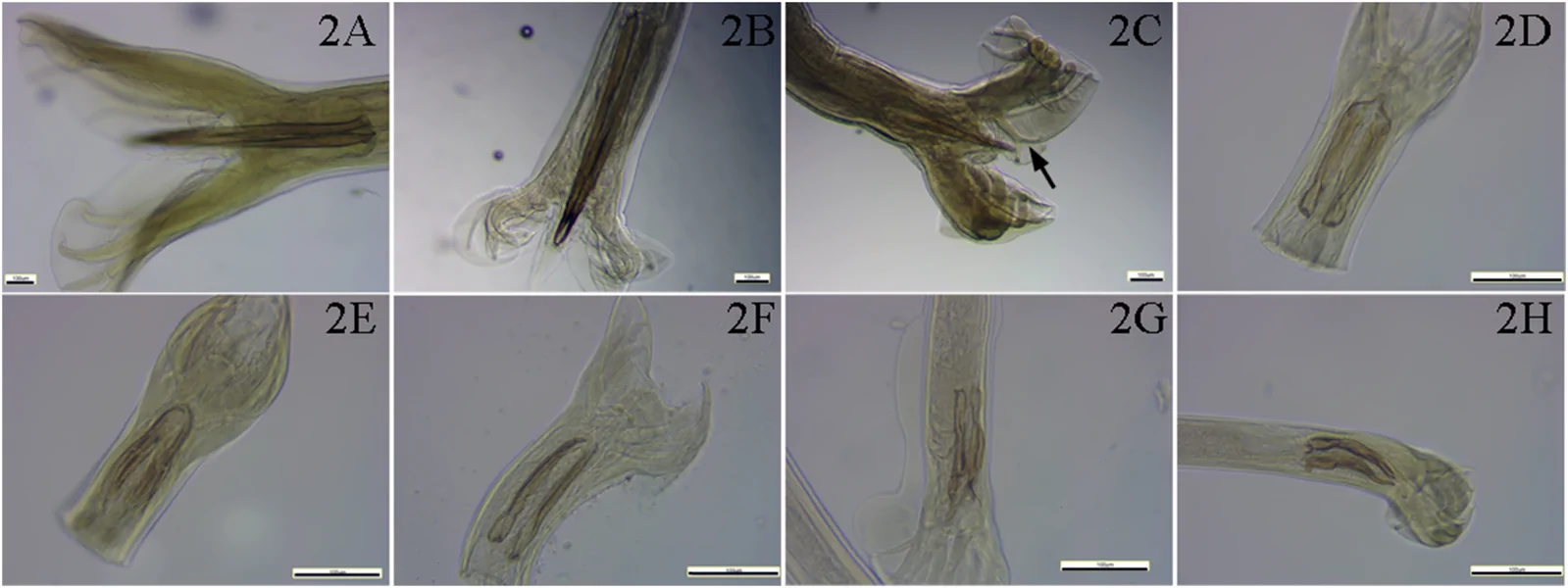
Male distal body parts of abomasal nematodes found in roe deer. (A) *Ashworthius sidemi* (×100) Bar = 100 μm; (B) *Ashworthius sidemi* (×100) Bar = 100 μm; (C) *Haemonchus contortus* (×100) Bar = 100 μm; arrow-dorsal ray; (D) *Mazamastrongylus dagestanica* (×200) Bar = 100 μm; (E) *Ostertagia kolchida* (×200) Bar = 100 μm; (F) *Ostertagia leptospicularis* (×200) Bar = 100 μm; (G) *Spiculopteragia boehmi* (×200) Bar = 100 μm; (H) *Trichostrongylus capricola* (×200) Bar = 100 μm.

Notably, the composition of abomasal nematode communities differed between regions. In the Rawa Mazowiecka region, only monoinfections with *H. contortus* were observed, whereas mixed infections comprising four and five species were recorded in the Węgrów and Iłża regions, respectively. The highest species richness was found in roe deer from the Iłża region, where the presence of the alien, blood-sucking species *A*. *sidemi* was recorded exclusively. No mixed infections with both Haemonchinae species, *H. contortus* and *A. sidemi*, were observed.

Additionally, *Spiculopteragia boehmi* was identified in animals from the Iłża region, together with *Ostertagia leptospicularis* and its minor morph, *O. kolchida*; however, these parasites were detected in only a single individual. Interestingly, *O. leptospicularis* was also found in a roe deer from the Węgrów region, where *Mazamastrongylus dagestanica* and *Trichostrongylus capricola* were also recorded.

### 3.2 Small intestine nematodes

In total, seven species of nematodes were identified in the small intestine of the examined roe deer (Table S2; Fig. 3). All species were detected in animals from the Iłża region, whereas lower species richness was observed in the two remaining areas. Representatives of the genera *Aonchotheca* and *Bunostomum* were found in the small intestine of animals from each location. A single *Nematodirus* nematode was detected exclusively in a roe deer from the Iłża region. Interestingly, *H. contortus*, which is typically located in the abomasum, as well as *Oe. venulosum* and *Ch. ovina*, typically found in the large intestine, were detected in the small intestine of some individuals. Both *Oe. venulosum* and *Ch. ovina* were confirmed by molecular analysis (GenBank: PV920951 and PV920952, respectively). The length of the obtained sequences ranged from 240 to 300 bp.

**Fig. 3:**
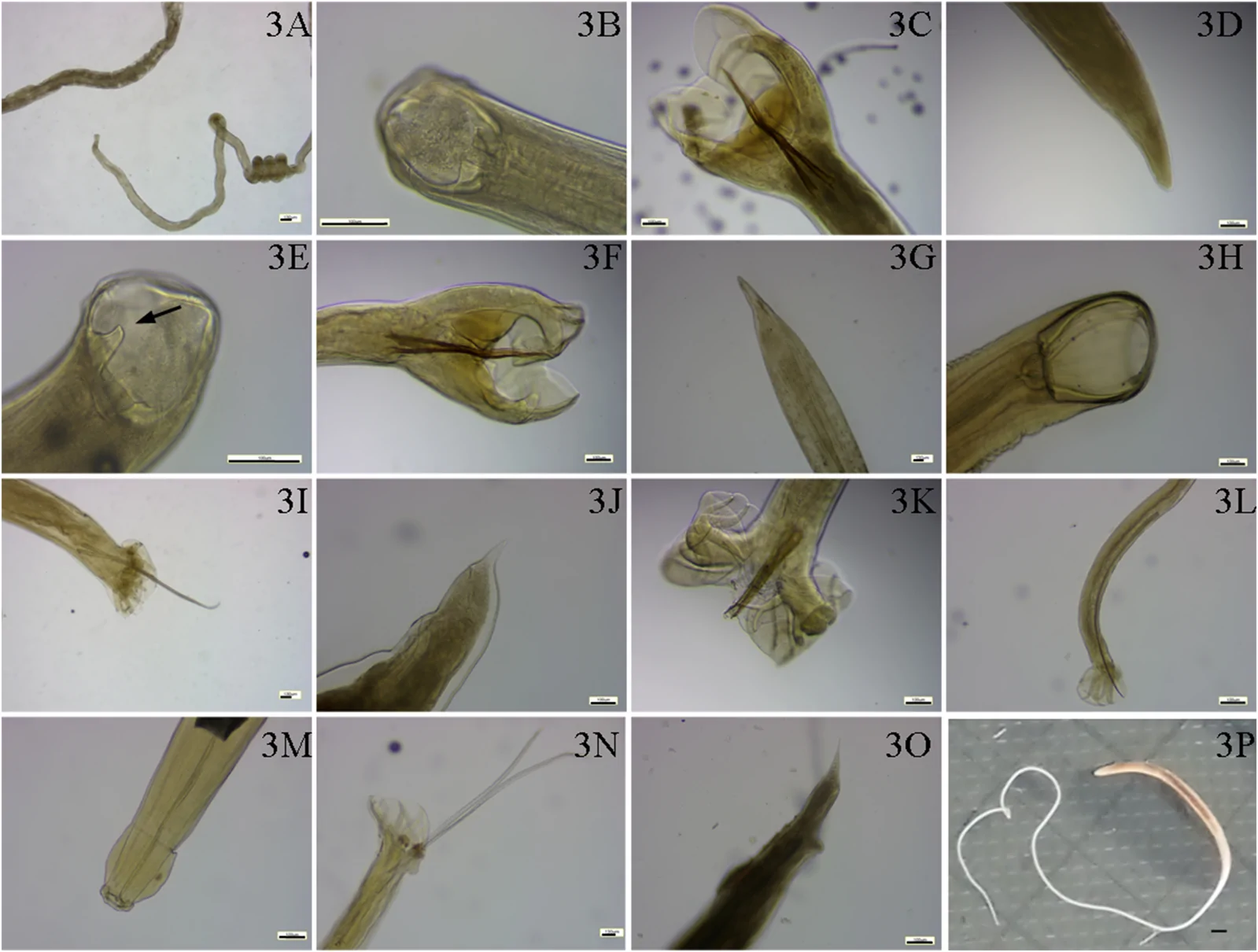
Nematodes found in the intestine of roe deer. (A) *Aonchotheca* sp. (×40) Bar = 130 μm; (B) *Bunostomum phlebotomum*, anterior end (×200) Bar = 100 μm; (C) *Bunostomum phlebotomum*, male posterior end (×100) Bar = 100 μm; (D) *Bunostomum phlebotomum*, female posterior end (×100) Bar = 100 μm; (E) *Bunostomum trigonocephalum*, anterior end (×200) Bar = 100 μm; (F) *Bunostomum trigonocephalum*, male posterior end (×100) Bar = 100 μm; arrow-dorsal tooth (G) *Bunostomum trigonocephalum*, female posterior end (×40) Bar = 130 μm; (H) *Chabertia ovina*, anterior end (×100) Bar = 100 μm; (I) *Chabertia ovina*, male posterior end (×40) Bar = 130 μm; (J) *Chabertia ovina*, female posterior end (×100) Bar = 100 μm; (K) *Haemonchus contortus* (×100) Bar = 100 μm; (L) *Nematodirus europaeus* (×100) Bar = 100 μm; (M) *Oesophagostomum venulosum*, anterior end (×100) Bar = 100 μm; (N) *Oesophagostomum venulosum*, male posterior end (×40) Bar = 130 μm; (O) *Oesophagostomum venulosum*, female posterior end (×100) Bar = 100 μm; (P) *Trichuris* sp. Bar = 20 mm.

Overall, *Aonchotheca* sp., *Ch. ovina*, *B. trigonocephalum*, and *Oe. venulosum* were commonly found in animals from all study areas. *Chabertia ovina* was the dominant species across all regions, with prevalence ranging from 15% in the Iłża region to 32% in the Węgrów region. Moreover, the highest infection intensity was also recorded for *Ch. ovina*, regardless of region, reaching a maximum of 66 individuals in a single host from the Węgrów region.

### 3.3 Small intestine cestodes

Fragments of tapeworm strobilae were found in the contents of the small intestine of four roe deer, including two animals from the Węgrów region and two from the Iłża region (prevalence: 11% and 4%, respectively) (Table S2).

Two partial cox1 sequences, each 396 bp long, were obtained from two cestodes, one collected in the Węgrów region and the other in the Iłża region (GenBank: PX511568 and PX511569, respectively). Although the isolates differed at the nucleotide level, they encoded identical amino acid sequences. The obtained sequences showed 87.6–93.5% identity with *Moniezia* spp. isolates from Denmark, China, India, Senegal, Ethiopia, Turkey, Viet Nam, and Taiwan, originating from both wild and domestic ruminants, including European bison, sheep, buffalo, cattle, and goats (Fig. 5). Due to the high level of variation within the analyzed cox1 fragment, species-level identification was not possible. Therefore, the specimens were classified as *Moniezia* sp.

### 3.4 Large intestine nematodes

A total of five nematode species were identified in the large intestine of roe deer (Table S3; Fig. 3). Nematodes such as *Ch. ovina*, *Oe. venulosum*, and *Trichuris* sp. were commonly observed regardless of the animals’ geographic origin. However, *Ch. ovina* was the dominant species across all locations, with a total prevalence of 69%. The highest intensity of *Ch. ovina* infection was recorded in a single individual from the Iłża region, in which 117 specimens were found. The mean length of *Ch*. *ovina* spicules was 1553 μm (range: 1324–1746 μm; n = 35). The fourth species, *B. trigonocephalum*, typically resides in the small intestine and was detected exclusively in one roe deer from the Rawa Mazowiecka region. Interestingly, *H. contortus*, the least prevalent species, was recorded in the cecum of a single animal from the Iłża region.

### 3.5 Prevalence of parasites in animals living on wind farms and outside wind farm areas

Regarding abomasum and small intestine nematodes, the prevalence of each species was higher in animals inhabiting areas within the wind farms, with one exception, which was the atypically located *Ch. ovina*. The same pattern applied to the large intestine nematodes, with one exception, including the nematodes of the genus *Trichuris*. Moreover, nematode species richness was consistently higher in hosts from areas within the wind farms. In particular, species composition was enriched by *M. dagestanica*, *O. kolchida*, *S. boehmi*, and *T. capricola* among abomasum nematodes (Table S4); *B. phlebotomum* and atypically located *H. contortus* among nematodes found in the small intestine (Table S5); and both atypically located: *B. trigonocephalum* and *H. contortus* among nematodes found in the large intestine (Table S6). Tapeworms of *Moniezia* sp. were also more prevalent in animals inhabiting wind farm areas compared to roe deer from areas outside the wind farms (Table S5).

### 3.6 Impact of selected factors on the number of nematodes in sections of the digestive tract (models 1-3)

For all sections of the digestive tract of roe deer, unique models that explained a significant proportion of the variability in nematode counts were obtained. However, not all best-fitting models for individual digestive tract sections included all variables as components. The presence of the wind farm significantly explained the variability in nematode counts in two of the three digestive tract sections: the abomasum and small intestine (Table S7). For the large intestine, this variable was excluded during model selection. However, a wind farm was present in candidate models within ΔAIC = 2. In these models, nematode numbers were higher in the farm area compared to the control areas. In both cases where the wind farm was a significant explanatory variable (for abomasum and small intestine), nematode counts were higher in the wind farm areas compared to the control areas (Fig. 4). The marginal mean in wind farm areas was approximately twice as high as in control areas. However, the standard error for individuals residing in wind farms was consistently greater (Fig. 4).

**Fig. 4:**
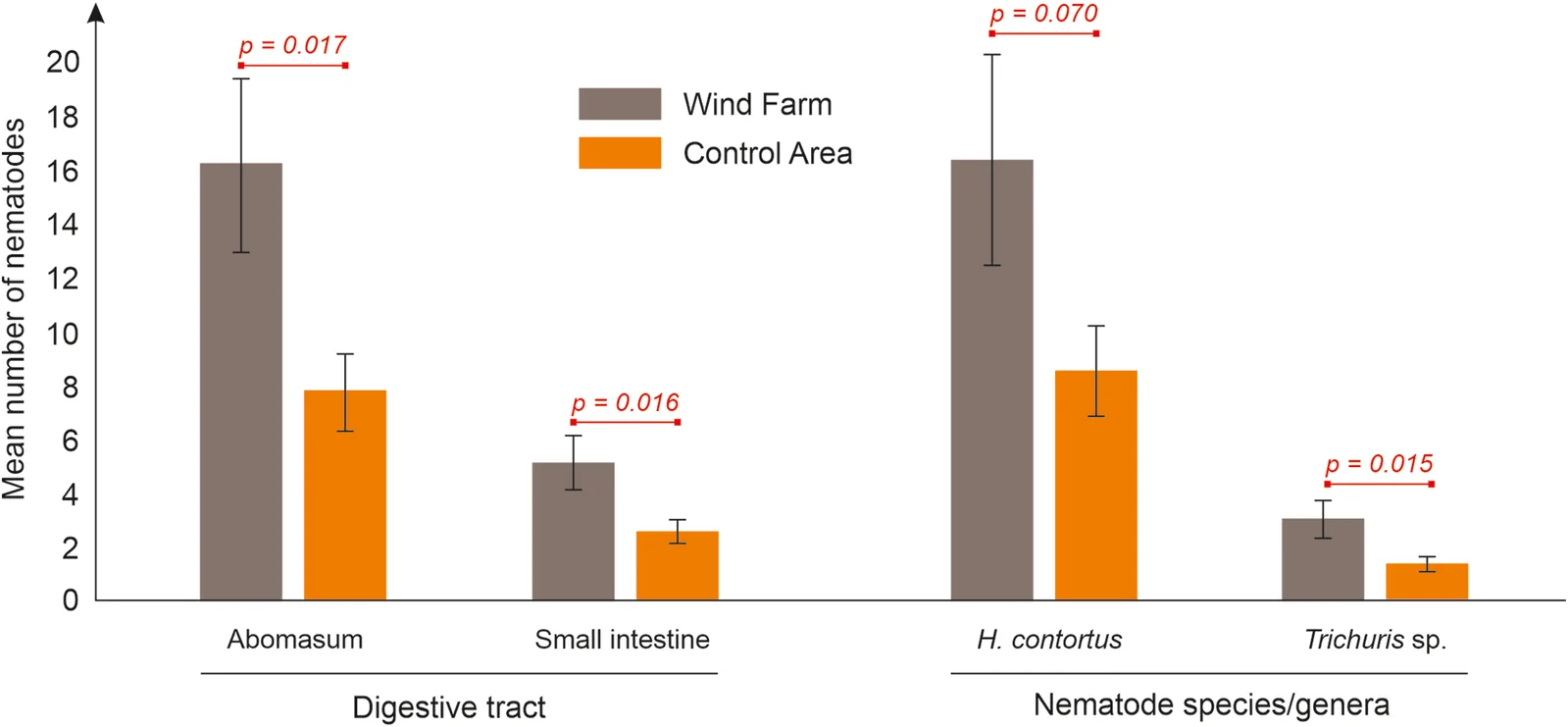
Mean numbers of nematodes in selected parts of the digestive tract in roe deer dwelling in wind farm areas and control areas. Marginal means calculated in generalized linear models with negative binomial distribution and log link function, based on the highest-ranked model composition. The figure also includes pairwise comparison of the means with the Least Significant Difference test in the same highest-ranked models.

**Fig. 5:**
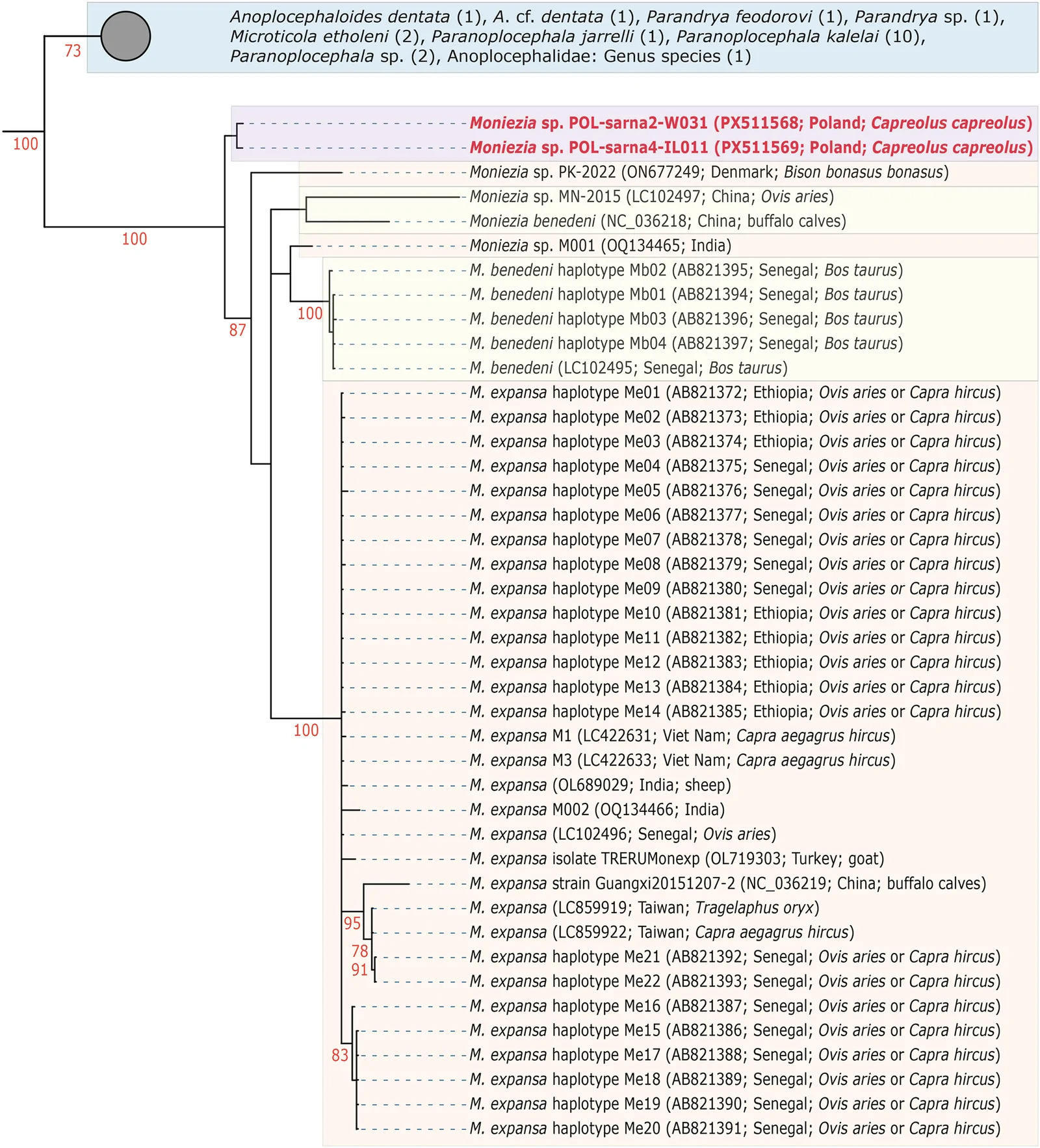
Bayesian inference tree based on the 370-bp fragment of 62 distinct sequences of the cox1 gene for cestodes of the family Anoplocephalidae (outgroup not shown). The number of sequences from each species is shown in parentheses. The Bayesian posterior probabilities ≥ 70% are shown adjacent to branch nodes. Sequences obtained in this study are shown in bold red.

The study area was an important variable in all best-fitting models for individual sections of the digestive tract, although not all comparisons between study areas were statistically significant (Table S7). Nevertheless, the study area's effect was unclear, as indicated by the varying estimates across study areas. Sex and age of animals were also important variables in the models, with sex appearing in all models and age in only two of three (exclusively in models for abomasum and small intestine). Regarding the sex of animals, females always showed lower nematode numbers than males (Table S7).

### 3.7 Impact of selected factors on the number of nematodes of selected species/genera in the whole digestive tract (models 4-7)

For all species/genera, unique models that significantly explained nematode count variability were obtained. However, not all best-fitting models for individual species/genus included all variables. The presence of the wind farm significantly explained the variability in nematode counts for two of the four species/genera: *H. contortus* and *Trichuris* sp. (Table S7). In the case of two others (*Ch*. *ovina* and *Oe. venulosum*), the wind farm was excluded during model selection. However, this explanatory variable was present in candidate models within ΔAIC = 2. In these models, nematode numbers were higher in the farm area compared to the control areas. In both cases where the wind farm was a significant explanatory variable (for *H. contortus* and *Trichuris* sp.), nematode counts were higher in the wind farm areas compared to the control areas (Fig. 4). The marginal mean in wind farm areas was approximately twice as high as in control areas. However, the standard error for individuals residing in wind farms was consistently greater (Fig. 4).

The study area was an important variable in two best-fitting models (for *H. contortus* and *Trichuris* sp.), although its effect was unclear, as indicated by the varying estimates across study areas (Table S7). Sex of animals appeared in three of four best-fitting models (except *Trichuris* sp.), and females always showed lower nematode numbers than males (Table S7). Age of animals was included in two of four best-fitting models, i.e., for *Ch. ovina* and *H. contortus*, but the effect was significant only for *H. contortus* and indicated an increase in the number of nematodes with age of roe deer (Table S7).

## 4 Discussion

The high diversity of GINs recorded in this study confirms that roe deer harbor complex parasite communities typical of European cervids. All GIN parasites identified here have previously been reported in this host (Demiaszkiewicz et al., 2017; Tomczuk et al., 2017; Macchioni et al., 2023; Pyziel-Serafin et al., 2023), although earlier data from Poland indicate an even greater species richness, including *O. antipini*, *O. lyrata*, *S. asymmetrica*, *S. mathevossiani*, *T. askivali*, and *T. axei* (Demiaszkiewicz et al., 2017; Pyziel-Serafin et al., 2023; Wyrobisz-Papiewska et al., 2025). Moreover, additional species have been reported in roe deer from other European countries and Russia, such as *Cooperia oncophora*, *Marshallagia marshalli*, *N*. *europaeus*, *N. filicollis*, *O. occidentalis*, *O. ostertagi*, and *T. colubriformis* (Hora et al., 2017; Kuznetsov et al., 2020; González et al., 2023; Macchioni et al., 2023). Together, these findings highlight both the richness and geographical variability of helminth communities in this host.

In contrast to nematodes, knowledge of cestode diversity in roe deer remains limited. To date, only two tapeworm species, *M. expansa* and *M. benedeni*, have been reported in Poland based on morphological identification (Pojmańska et al., 2007; Tomczuk et al., 2017). The detection of *Moniezia* sp. with low genetic similarity to reference sequences in the present study supports earlier suggestions that insufficient molecular data hinder accurate species identification in wild ruminants (Avramenko et al., 2018). This finding points to potential cryptic diversity within the genus and suggests the presence of a third, previously unrecognized *Moniezia* species in Poland, emphasizing the need for integrative taxonomic approaches combining molecular and morphological methods. Similarly, the inability to identify *Aonchotheca* and *Trichuris* to species level in this study is consistent with previous reports of *T. globulosa* and *A. bovis* (syn. *Capillaria bovis*) in roe deer in Poland (Tomczuk et al., 2017).

Particular attention should be paid to the occurrence of hematophagous nematodes of the subfamily Haemonchinae. Both *A. sidemi* and *H. contortus* were recorded in the present study, in agreement with some previous reports (Wyrobisz-Papiewska et al., 2025), but contrasting with others that reported the dominance of only one of these species (Demiaszkiewicz et al., 2017; Tomczuk et al., 2017; Kuznetsov et al., 2020; González et al., 2023; Pyziel-Serafin et al., 2023). The dominance of *H. contortus* observed here is consistent with previous reports suggesting an increasing importance of this species in wildlife populations. Although parasite transmission between wild and domestic ruminants was not directly assessed in the present study, the observed pattern may be consistent with potential parasite exchange, particularly in areas where grazing habitats overlap (Morgan et al., 2013). Interestingly, *H. contortus* and *B. trigonocephalum* were found in the large intestine of a single roe deer, although they typically reside in the abomasum and small intestine, respectively. Additionally, large intestine nematodes, such as *Ch. ovina* and *Oe. venulosum*, were detected in the small intestine. In that section of the digestive tract, *Ch. ovina* was the main component of the helminth fauna in the examined roe deer. A similar observation was made previously, as the presence of *N. helvetianus*, *A. sidemi,* and *B. trigonocephalum* was noted in the large intestine of European bison, as a result of their high intensity of infection and dispersion to further parts of the digestive tract of a host (Demiaszkiewicz et al., 2012). The parasite assemblage identified in this study includes numerous species with well-documented pathogenic effects. Hematophagous nematodes such as *H. contortus* and *A. sidemi* can cause severe anemia and reduced fitness (Kaplan et al., 2004), while abomasal species, including *O*. *kolchida*, *O. leptospicularis*, *M*. *dagestanica*, and *S*. *boehmi,* may induce gastric disturbances and weight loss (Soulsby, 1982). Intestinal nematodes such as *Ch. ovina*, *Oe*. *venulosum*, *N*. *europaeus*, and *Trichuris* sp. are associated with enteritis and secondary infections (Zajac and Conboy, 2012). In addition, hookworms (*B*. *phlebotomum*, *B. trigonocephalum*) contribute to anemia, whereas less pathogenic taxa, including *Aonchotheca* sp. and *Moniezia* sp., may still affect host condition under heavy infection. Importantly, mixed infections are likely to have synergistic effects, further exacerbating negative impacts on host health (Kaplan et al., 2004; Zajac and Conboy, 2012).

One of the most significant findings of this study is the effect of wind farm presence on parasite infections. Roe deer inhabiting wind farm areas exhibited generally higher nematode abundance and species richness, suggesting that anthropogenic infrastructure can influence host-parasite interactions. This, to our knowledge, represents the first data linking wind farms to gastrointestinal helminth dynamics in terrestrial mammals. It is worth noting that other factors not accounted for in this study may have been present and ultimately modulated the observed effect. Previous research in such environments has focused mainly on behavioral and ecological responses (Flydal et al., 2004; Walter et al., 2006; Skarin et al., 2015; Kim et al., 2025), with some studies indicating that cumulative stressors associated with wind farms may negatively affect fitness and survival (Taylor et al., 2016; Milligan et al., 2021). Supporting this interpretation, anthropogenic disturbance has been shown to induce physiological stress responses, including elevated glucocorticoid levels (Klich et al., 2020; Teff-Seker et al., 2022), as well as reduced body condition (Karwowska et al., 2015; Agnew et al., 2016; Łopucki et al., 2018), and developmental abnormalities (Klich et al., 2024). Since chronic stress can impair immune function (Sapolsky et al., 2000), it is plausible that such mechanisms contribute to increased susceptibility to parasitic infections. Although direct evidence linking these processes to disease dynamics has been limited (Köppel, 2017), the present study suggests that they may result in measurable differences in parasite burden.

It should be noted that the effect of wind farm presence was not always reflected in statistically significant differences in nematode counts, although this variable was consistently retained in the most strongly supported models. This finding suggests that wind farm presence contributes to explaining variation in nematode abundance in roe deer. The lack of a clear effect in some analyses may result from behavioral compensation by roe deer. Previous studies have shown that roe deer tend to avoid areas immediately surrounding turbines, resulting in altered spatial distribution patterns in wind farm landscapes (Gmaj and Klich, 2026). Consequently, some individuals classified as inhabiting wind farm areas may have used these habitats only temporarily. This interpretation is supported by the relatively large standard errors observed for roe deer sampled within wind farm areas compared with those from control areas. However, the mechanisms underlying the observed association were not directly investigated in the present study and therefore remain speculative. Together, these findings highlight the complex interactions among environmental factors, host condition, and parasite ecology. Our study did not provide a clear answer on whether the wind farm infrastructure was a direct driver of the observed differences or whether there were other confounding effects we could not observe. Therefore, further research is needed to determine the mechanisms linking wind farm infrastructure and gastrointestinal helminth infections in roe deer.

## CRediT authorship contribution statement

**Anna Maria Pyziel:** Writing – original draft, Visualization, Validation, Methodology, Investigation, Formal analysis, Data curation, Conceptualization. **Kateryna Slivinska:** Writing – review & editing, Investigation. **Rusłan Sałamatin:** Writing – review & editing, Investigation. **Joanna Werszko:** Writing – review & editing, Investigation. **Marta Kloch:** Writing – review & editing, Investigation. **Marcin Świątek:** Writing – review & editing, Investigation. **Olena Zhytova:** Writing – review & editing, Investigation. **Daniel Klich:** Writing – original draft, Supervision, Project administration, Methodology, Investigation, Funding acquisition, Formal analysis, Data curation, Conceptualization.

## Financial disclosure statement

The study was partially financed by the National Science Centre, Poland (Grant number: 2021/41/B/NZ9/04442), and by the European Union (MSCA4Ukraine project https://cordis.europa.eu/project/id/101101923). Views and opinions expressed are, however, those of the author(s) only and do not necessarily reflect those of the European Union. Neither the European Union nor the MSCA4Ukraine Consortium as a whole nor any individual member institutions of the MSCA4Ukraine Consortium can be held responsible for them.

## Animal rights statement

None required, as the samples were taken exclusively from animals legally hunted during the hunting seasons 2022 and 2024 according to Polish hunting law (Act of the Polish Parliament dated 13 October 1995, item 713, the Hunting Law).

## Declaration of competing interest

The authors declare the following financial interests/personal relationships which may be considered as potential competing interests: Daniel Klich reports financial support was provided by National Science Centre Poland. Kateryna Slivinska reports financial support was provided by European Commission. The other authors declare that they have no known competing financial interests or personal relationships that could have appeared to influence the work reported in this paper.
