# Supplementary material for: Do wind energy installations alter gastrointestinal parasite infection patterns in roe deer (Capreolus capreolus L.) in central Poland?
Source: Int J Parasitol Parasites Wildl. 2026 Oct 8;31:101300. doi: 10.1016/j.ijppaw.2026.101300 (PMC13682463; doi:10.1016/j.ijppaw.2026.101300)
Supplement: Supplementary file 1 [file mmc1.docx]

Table S1. Prevalence with confidence intervals [P (CI95%)] and intensity of infection [Range and mean (X)] with abomasal nematodes in roe deer from Central Poland

| Nematode species | Iłża (*n*=48) | | | Rawa Mazowiecka (*n*=31) | | | Węgrów (*n*=19) | | | Total (*n*=98) | | |
| --- | --- | --- | --- | --- | --- | --- | --- | --- | --- | --- | --- | --- |
|  | P (CI 95%) | Range | X | P (CI 95%) | Range | X | P (CI 95%) | Range | X | P (CI 95%) | Range | X |
| *A. sidemi* | 0.06 (0.01-0.17) | 2-47 | 20 | 0 | - | - | 0 | - | - | 0.03 (0.01-0.09) | 2-74 | 29 |
| *H. contortus* | 0.35 (0.22-0.51) | 1-1019 | 161.5 | 0.45 (0.27-0.64) | 1-143 | 23.8 | 0.53 (0.29-0.76) | 9-2558 | 343.3 | 0.42 (0.32-0.52) | 1-2558 | 157.5 |
| *M. dagestanica* | 0 | - | - | 0 | - | - | 0.05 (0.00-0.26) | 2-2 | 2 | 0.01 (0.00-0.06) | 2-2 | 2 |
| *O. kolchida* | 0.02 (0.00-0.11) | 1-1 | 1 | 0 | - | - | 0 | - | - | 0.01 (0.00-0.06) | 1-1 | 1 |
| *O. leptospicularis* | 0.02 (0.00-0.11) | 1-1 | 1 | 0 | - | - | 0.05 (0.00-0.26) | 1-1 | 1 | 0.02 (0.00-0.07) | 1-1 | 1 |
| *S. boehmi* | 0.02 (0.00-0.11) | 1-1 | 1 | 0 | - | - | 0 | - | - | 0.01 (0.00-0.06) | 1-1 | 1 |
| *T. capricola* | 0 | - | - | 0 | - | - | 0.05 (0.00-0.26) | 1-1 | 1 | 0.01 (0.00-0.06) | 1-1 | 1 |
