# Supplementary material for: Do wind energy installations alter gastrointestinal parasite infection patterns in roe deer (Capreolus capreolus L.) in central Poland?
Source: Int J Parasitol Parasites Wildl. 2026 Oct 8;31:101300. doi: 10.1016/j.ijppaw.2026.101300 (PMC13682463; doi:10.1016/j.ijppaw.2026.101300)
Supplement: Supplementary file 2 [file mmc2.docx]

Table S2. Prevalence with confidence intervals [P (CI95%)] and intensity of infection [Range and mean (X)] with small intestine helminths in roe deer from Central Poland

| Helminth | Iłża (*n*=46) | | | Rawa Mazowiecka (*n*=33) | | | Węgrów (*n*=19) | | | Total (*n*=98) | | |
| --- | --- | --- | --- | --- | --- | --- | --- | --- | --- | --- | --- | --- |
|  | P (CI 95%) | Range | X | P (CI 95%) | Range | X | P (CI 95%) | Range | X | P (CI 95%) | Range | X |
| *Aonchotheca* sp. | 0.15 (0.06-0.27) | 1-65 | 10.7 | 0.12 (0.03-0.38) | 7-36 | 15.2 | 0.21 (0.06-0.46) | 1-3 | 2 | 0.15 (0.09-0.24) | 1-65 | 9.6 |
| *B. phlebotomum* | 0.02 (0.00-0.12) | 3-3 | 3 | 0 | - | - | 0 | - | - | 0.01 (0.00-0.06) | 3-3 | 3 |
| *B. trigonocephalum* | 0.04 (0.01-0.15) | 2-2 | 2 | 0.09 (0.02-0.24) | 1-5 | 2.7 | 0.05 (0.00-0.26) | 2-2 | 2 | 0.06 (0.02-0.13) | 1-5 | 2.3 |
| *Ch. ovina* | 0.15 (0.06-0.29) | 1-66 | 14.3 | 0.24 (0.11-0.42) | 1-10 | 2.7 | 0.32 (0.13-0.57) | 2-30 | 12.8 | 0.21 (0.14-0.31) | 1-66 | 9.5 |
| *H. contortus* | 0.07 (0.01-0.18) | 1.7 | 1-2 | 0 | - | - | 0 | - | - | 0.03 (0.01-0.09) | 1-2 | 1.7 |
| *Moniezia* sp. | 0.04 (0.01-0.15) | 1-1 | 1 | 0 | - | - | 0.11 (0.01-0.33) | 1-1 | 1 | 0.04 (0.01-0.10) | 1-1 | 1 |
| *N. europeus* | 0.02 (0.00-0.12) | 1-1 | 1 | 0 | - | - | 0 | - | - | 0.02 (0.00-0.07) | 1-1 | 1 |
| *Oe. venulosum* | 0.04 (0.01-0.15) | 1-3 | 2 | 0.09 (0.02-0.24) | 1-1 | 1 | 0.05 (0.00-0.26) | 1-1 | 1 | 0.05 (0.02-0.12) | 1-3 | 1.3 |
