# Supplementary material for: Do wind energy installations alter gastrointestinal parasite infection patterns in roe deer (Capreolus capreolus L.) in central Poland?
Source: Int J Parasitol Parasites Wildl. 2026 Oct 8;31:101300. doi: 10.1016/j.ijppaw.2026.101300 (PMC13682463; doi:10.1016/j.ijppaw.2026.101300)
Supplement: Supplementary file 3 [file mmc3.docx]

Table S3. Prevalence with confidence intervals [P (CI95%)] and intensity of infection [Range and mean (X)] with large intestine nematodes in roe deer from Central Poland

| Nematode species | Iłża (*n*=43) | | | Rawa Mazowiecka (*n*=23) | | | Węgrów (*n*=19) | | | Total (*n*=85) | | |
| --- | --- | --- | --- | --- | --- | --- | --- | --- | --- | --- | --- | --- |
|  | P (CI 95%) | Range | X | P (CI 95%) | Range | X | P (CI 95%) | Range | X | P (CI 95%) | Range | X |
| *B. trigonocephalum* | 0 | - | - | 0.04 (0.00-0.22) | 1-1 | 1 | 0 | - | - | 0.01 (0.00-0.06) | 1-2 | 1.5 |
| *Ch. ovina* | 0.72 (0.56-0.85) | 1-117 | 23 | 0.70 (0.47-0.87) | 1-66 | 20.8 | 0.63 (0.38-0.84) | 2-43 | 11.6 | 0.69 (0.58-0.79) | 1-117 | 20.1 |
| *H. contortus* | 0.02 (0.00-0.12) | 2-2 | 2 | 0 | - | - | 0 | - | - | 0.01 (0.00-0.06) | 2-2 | 2 |
| *Oe. venulosum* | 0.33 (0.19-0.49) | 1-25 | 9.4 | 0.30 (0.13-0.53) | 1-14 | 7.4 | 0.47 (0.24-0.71) | 2-12 | 6.2 | 0.35 (0.25-0.46) | 1-25 | 8 |
| *Trichuris* sp. | 0.58 (0.42-0.73) | 1-46 | 7.6 | 0.48 (0.27-0.69) | 1-26 | 5.9 | 0.37 (0.16-0.62) | 1-13 | 3.7 | 0.51 (0.40-0.62) | 1-46 | 6.5 |
