# Supplementary material for: Do wind energy installations alter gastrointestinal parasite infection patterns in roe deer (Capreolus capreolus L.) in central Poland?
Source: Int J Parasitol Parasites Wildl. 2026 Oct 8;31:101300. doi: 10.1016/j.ijppaw.2026.101300 (PMC13682463; doi:10.1016/j.ijppaw.2026.101300)
Supplement: Supplementary file 4 [file mmc4.docx]

Table S4. Comparison between the species composition and prevalence [P (CI 95%)] of infection with abomasal nematodes of roe deer inhabiting areas outside the wind farms (C – control), and inside the wind farms (WF – Wind farm) in Central Poland

| Nematode | Iłża (*n*=48) | | Rawa Mazowiecka (*n*=31) | | Węgrów (*n*=19) | | Total (*n*=98) | |
| --- | --- | --- | --- | --- | --- | --- | --- | --- |
|  | C (*n*=30) | WF (*n*=18) | C (*n*=21) | WF (*n*=10) | C (*n*=11) | WF (*n*=8) | C (*n*=62) | WF (*n*=36) |
| *A. sidemi* | 0.03 (0.00-0.17) | 0.11 (0.01-0.35) | 0 | 0 | 0 | 0 | 0.02 (0.00-0.09) | 0.06 (0.01-0.19) |
| *H. contortus* | 0.20 (0.08-0.39) | 0.61 (0.36-0.83) | 0.38 (0.18-0.62) | 0.50 (0.19-0.81) | 0.54 (0.23-0.83) | 0.50 (0.16-0.84) | 0.32 (0.21-0.45) | 0.56 (0.38-0.72) |
| *M. dagestanica* | 0 | 0 | 0 | 0 | 0 | 0.13 (0.00-0.53) | 0 | 0.03 (0.00-0.15) |
| *O. kolchida* | 0 | 0.06 (0.00-0.27) | 0 | 0 | 0 | 0 | 0 | 0.03 (0.00-0.15) |
| *O. leptospicularias* | 0 | 0 | 0 | 0 | 0 | 0.13 (0.00-0.53) | 0.02 (0.00-0.09) | 0.03 (0.00-0.15) |
| *S. boehmi* | 0 | 0.06 (0.00-0.27) | 0 | 0 | 0 | 0 | 0 | 0.03 (0.00-0.15) |
| *T. capricola* | 0 | 0 | 0 | 0 | 0 | 0.13 (0.00-0.53) | 0 | 0.03 (0.00-0.15) |
