# Supplementary material for: Do wind energy installations alter gastrointestinal parasite infection patterns in roe deer (Capreolus capreolus L.) in central Poland?
Source: Int J Parasitol Parasites Wildl. 2026 Oct 8;31:101300. doi: 10.1016/j.ijppaw.2026.101300 (PMC13682463; doi:10.1016/j.ijppaw.2026.101300)
Supplement: Supplementary file 5 [file mmc5.docx]

Table S5. Comparison between the species composition and prevalence [P (CI 95%)] of infection with small intestine helminths of roe deer inhabiting areas outside the wind farms (C – control), and inside the wind farms (WF – Wind farm) in Central Poland

| Helminth | Iłża (*n*=46) | | Rawa Mazowiecka (*n*=33) | | Węgrów (*n*=19) | | Total *(n*=98) | |
| --- | --- | --- | --- | --- | --- | --- | --- | --- |
|  | C (*n*=29) | WF (*n*=17) | C (*n*=21) | WF (*n*=12) | C (*n*=10) | WF (*n*=9) | C (*n*=60) | WF (*n*=38) |
| *Aonchoteca* sp. | 0.14 (0.04-0.32) | 0.18 (0.04-0.43) | 0.05 (0.00-0.24) | 0.25 (0.05-0.57) | 0.10 (0.00-0.44) | 0.33 (0.07-0.70) | 0.10 (0.04-0.20) | 0.24 (0.11-0.40) |
| *B. phlebotomum* | 0 | 0.06 (0.00-0.29) | 0 | 0 | 0 | 0 | 0 | 0.03 (0.00-0.14) |
| *B. trigonocephalum* | 0 | 0.06 (0.00-0.29) | 0.05 (0.00-0.24) | 0.17 (0.02-0.48) | 0.10 (0.00-0.44) | 0 | 0.03 (0.00-0.12) | 0.08 (0.02-0.21) |
| *Ch. ovina* | 0.10 (0.02-0.27) | 0.24 (0.07-0.50) | 0.29 (0.11-0.52) | 0.08 (0.00-0.38) | 0.40 (0.12-0.74) | 0.22 (0.03-0.60) | 0.22 (0.12-0.34) | 0.18 (0.08-0.34) |
| *H. contortus* | 0 | 0.12 (0.01-0.36) | 0 | 0 | 0 | 0 | 0 | 0.05 (0.01-0.18) |
| *Moniezia* sp. | 0.03 (0.00-0.18) | 0.06 (0.00-0.29) | 0 | 0 | 0 | 0.22 (0.03-0.60) | 0.02 (0.00-0.09) | 0.08 (0.02-0.21) |
| *N. europeus* | 0.03 (0.00-0.18) | 0 | 0 | 0.08 (0.00-0.38) | 0 | 0 | 0.02 (0.00-0.09) | 0.03 (0.00-0.14) |
| *Oe. venulosum* | 0 | 0.12 (0.01-0.36) | 0.10 (0.01-0.30) | 0.08 (0.00-0.38) | 0 | 0.11 (0.00-0.48) | 0.03 (0.00-0.12) | 0.11 (0.03-0.25) |
