# Supplementary material for: Do wind energy installations alter gastrointestinal parasite infection patterns in roe deer (Capreolus capreolus L.) in central Poland?
Source: Int J Parasitol Parasites Wildl. 2026 Oct 8;31:101300. doi: 10.1016/j.ijppaw.2026.101300 (PMC13682463; doi:10.1016/j.ijppaw.2026.101300)
Supplement: Supplementary file 6 [file mmc6.docx]

Table S6. Comparison between the species composition and prevalence [P (CI 95%)] of infection with large intestine nematodes of roe deer inhabiting areas outside the wind farms (C – control), and inside the wind farms (WF – Wind farm) in Central Poland

| Nematode | Iłża (*n*=43) | | Rawa Mazowiecka (*n*=23) | | Węgrów (*n*=10) | | Total (*n*=85) | |
| --- | --- | --- | --- | --- | --- | --- | --- | --- |
|  | C (*n*=27) | WF (*n*=16) | C (*n*=15) | WF (*n*=8) | C (*n*=10) | WF (*n*=9) | C (*n*=52) | WF (*n*=33) |
| *B. trigonocephalum* | 0 | 0 | 0 | 0.12 (0.00-0.53) | 0 | 0 | 0 | 0.03 (0.00-0.16) |
| *Ch. ovina* | 0.74 (0.54-0.89) | 0.69 (0.41-0.89) | 0.53 (0.27-0.79) | 1.0 (0.63-1) | 0.60 (0.26-0.88) | 0.67 (0.30-0.93) | 0.65 (0.51-0.78) | 0.75 (0.58-0.89) |
| *H. contortus* | 0 | 0.06 (0.00-0.30) | 0 | 0 | 0 | 0 | 0 | 0.03 (0.00-0.16) |
| *Oe. venulosum* | 0.22 (0.09-0.42) | 0.50 (0.25-0.75) | 0.27 (0.08-0.55) | 0.38 (0.09-0.76) | 0.40 (0.12-0.73) | 0.56 (0.21-0.86) | 0.27 (0.16-0.41) | 0.48 (0.31-0.66) |
| *Trichuris* sp | 0.59 (0.39-0.78) | 0.56 (0.30-0.80) | 0.47 (0.21-0.73) | 0.50 (0.16-0.84) | 0.50 (0.19-0.81) | 0.22 (0.03-0.60) | 0.54 (0.39-0.68) | 0.45 (0.28-0.64) |
