# Supplementary material for: Do wind energy installations alter gastrointestinal parasite infection patterns in roe deer (Capreolus capreolus L.) in central Poland?
Source: Int J Parasitol Parasites Wildl. 2026 Oct 8;31:101300. doi: 10.1016/j.ijppaw.2026.101300 (PMC13682463; doi:10.1016/j.ijppaw.2026.101300)
Supplement: Supplementary file 7 [file mmc7.docx]

Table. S7. Effects of wind farm (WF), study area (AREA), sex (SEX), and age (AGE) of animals on the number of nematodes in selected parts of the digestive tract (Models 1-3) and selected nematode species in whole digestive tract (Models 4-7 ) in roe deer in separate generalized linear models; variables not included in the table for the given section of the digestive tract were excluded during model selection

| Parameter | Estimate | St. error | Lower CI | Upper CI | Wald Chi^2^ | P-value |
| --- | --- | --- | --- | --- | --- | --- |
| ***Model 1: Abomasum*** | | | | | | |
| Intercept | 1.306 | 0.724 | -0.113 | 2.725 | 3.255 | 0.071 |
| WF (yes) | 0.733 | 0.272 | 0.200 | 1.265 | 7.277 | 0.007 |
| WF (no) | 0* |  |  |  |  |  |
| AREA (Iłża) | -0.799 | 0.306 | -1.399 | -0.200 | 8.827 | 0.009 |
| AREA (Rawa M.) | -0.961 | 0.412 | -1.768 | -0.153 | 5.437 | 0.020 |
| AREA (Węgrów) | 0* |  |  |  |  |  |
| SEX (F) | -2.907 | 0.365 | -3.623 | -2.190 | 63.267 | <0.001 |
| SEX (M) | 0* |  |  |  |  |  |
| AGE | 0.700 | 0.132 | 0.442 | 0.959 | 28.242 | <0.001 |
| ***Model 2: Small intestine*** | | | | | | |
| Intercept | 2.842 | 0.508 | 1.847 | 3.837 | 31.331 | <0.001 |
| WF (yes) | 0.671 | 0.241 | 0.198 | 1.144 | 7.735 | 0.005 |
| WF (no) | 0* |  |  |  |  |  |
| AREA (Iłża) | -0.329 | 0.316 | -0.948 | 0.291 | 1.083 | 0.298 |
| AREA (Rawa M.) | -0.754 | 0.345 | -1.430 | -0.078 | 4.774 | 0.029 |
| AREA (Węgrów) | 0* |  |  |  |  |  |
| AGE | -0.382 | 0.105 | -0.588 | -0.176 | 13.204 | <0.001 |
| ***Model 3: Large intestine*** | | | | | | |
| Intercept | 3.705 | 0.550 | 2.627 | 4.783 | 45.402 | <0.001 |
| AREA (Iłża) | 0.905 | 0.303 | 0.312 | 1.498 | 8.940 | 0.003 |
| AREA (Rawa M.) | 0.705 | 0.323 | 0.063 | 1.348 | 4.628 | 0.031 |
| AREA (Węgrów) | 0* |  |  |  |  |  |
| SEX (F) | -1.345 | 0.309 | -1.950 | -0.739 | 18.953 | <0.001 |
| SEX (M) | 0* |  |  |  |  |  |
| AGE | -0.228 | 0.108 | -0.438 | -0.017 | 4.478 | 0.034 |
| ***Model 4: Chabertia sp.*** | | | | | | |
| Intercept | 3.899 | 0.534 | 2.854 | 4.945 | 53.403 | <0.001 |
| SEX (F) | -1.216 | 0.291 | -1.786 | -0.646 | 17.469 | <0.001 |
| SEX (M) | 0* |  |  |  |  |  |
| AGE | -0.178 | 0.106 | -0.385 | 0.028 | 2.858 | 0.091 |
| ***Model 5: Haemonchus contortus*** | | | | | | |
| Intercept | 0.709 | 0.811 | -0.879 | 2.298 | 0.766 | 0.382 |
| WF (yes) | 0.647 | 0.313 | 0.034 | 1.260 | 4.279 | 0.039 |
| WF (no) | 0* |  |  |  |  |  |
| AREA (Iłża) | -0.984 | 0.322 | -1.615 | -0.353 | 9.345 | 0.002 |
| AREA (Rawa M.) | -0.925 | 0.442 | -1.791 | -0.059 | 4.387 | 0.036 |
| AREA (Węgrów) | 0* |  |  |  |  |  |
| SEX (F) | -2.871 | 0.410 | -3.585 | -1.977 | 45.936 | <0.001 |
| SEX (M) | 0* |  |  |  |  |  |
| AGE | 0.853 | 0.148 | -0.562 | 1.143 | 33.113 | <0.001 |
| ***Model 6: Oesophagostomum sp.*** | | | | | | |
| Intercept | 1.494 | 0.167 | 1.167 | 1.821 | 80.196 | <0.001 |
| SEX (F) | -2.960 | 0.406 | -3.755 | -2.165 | 53.245 | <0.001 |
| SEX (M) | 0* |  |  |  |  |  |
| ***Model 7: Trichuris sp.*** | | | | | | |
| Intercept | -0.637 | 0.387 | -1.395 | 0.121 | 2.714 | 0.099 |
| WF (yes) | 0.780 | 0.270 | 0.251 | 1.308 | 8.371 | 0.004 |
| WF (no) | 0* |  |  |  |  |  |
| AREA (Iłża) | 1.613 | 0.411 | 0.808 | 2.418 | 15.422 | <0.001 |
| AREA (Rawa M.) | 1.274 | 0.443 | 0.406 | 2.142 | 8.282 | 0.004 |
| AREA (Węgrów) | 0* |  |  |  |  |  |
